# Resveratrol inhibits the malignant progression of hepatocellular carcinoma via MARCH1-induced regulation of PTEN/AKT signaling

**DOI:** 10.18632/aging.103338

**Published:** 2020-06-12

**Authors:** Hanhan Dai, Minjing Li, Wei Yang, Xiucui Sun, Peiyuan Wang, Xia Wang, Jiaqi Su, Xu Wang, Xuemei Hu, Mingdong Zhao

**Affiliations:** 1 Department of Imaging, Binzhou Medical University, Yantai 264003, Shandong, PR China; 2 Department of Immunology, Binzhou Medical University, Yantai 264003, Shandong, PR China; 3 Department of Chinese medicine prescription, Binzhou Medical University, Yantai 264003, Shandong, PR China; 4 Department of Oral Pathology, Binzhou Medical University, Yantai 264003, Shandong, PR China

**Keywords:** hepatocellular carcinoma, resveratrol, survival, MARCH1, PTEN/AKT

## Abstract

Resveratrol is a common, naturally occurring polyphenol confirmed with inhibited the cellular effects of carcinogenesis. However, the molecular mechanism underlying resveratrol’s action against hepatocellular carcinoma (HCC) is still unclear. In addition, MARCH1 promotes the initiation and progression of HCC, but it is unclear whether resveratrol exerts antitumor efforts by regulating MARCH1 expression. This study determined the molecular mechanisms underlying the antitumor effects of resveratrol in HCC. Resveratrol induced apoptosis and inhibited the proliferation, migration, and invasion of HCC cell lines (HepG2 and Hep3B). In addition, it inhibited MARCH1 and phospho–protein kinase B (p-AKT) expression but upregulated the phosphatase and tensin homolog deleted on chromosome 10 (PTEN) dose-dependently both in vitro and in vivo. MARCH1 knockdown by small interfering RNA (siRNA) also increased PTEN expression. Meanwhile, MK2206 (an AKT inhibitor) and bisperoxovanadium (BPV; a PTEN inhibitor) combined with resveratrol decreased MARCH1 expression more than the single-treatment HCC group. These results suggested that resveratrol affects the biological characteristics of HCC via downregulation of MARCH1 expression.

## INTRODUCTION

Liver cancer is a leading cause of cancer burden globally. There are approximately 841,000 new cases and 782,000 deaths have been reported annually, making it has emerged as the sixth most commonly diagnosed cancer and fourth most deadly type of cancer worldwide in 2018. Hepatocellular carcinoma (HCC) is the main form of primary liver cancer (comprising 75%–85% of cases) [1]. Unfortunately, HCC is usually diagnosed at an advanced stage, and the 5-year survival rate is extremely low (~15%) [2]. Although many researchers are trying to exploit molecular targeted drugs and immunotherapies, effective agents for HCC treatment have not yet been developed [3]. Therefore, there is an urgent need for effective therapeutic agents for HCC treatment.

Resveratrol is a natural polyphenolic compound widely available from both dietary foods and plant species. Studies have reported that resveratrol has many health benefits, such as antioxidant, anti-inflammatory, antiviral, anti-hyperlipidemia activities [4]. Increasing evidence indicates that resveratrol shows significant effects against different types of cancer. For example, resveratrol inhibits oral squamous cell carcinoma via induction of apoptosis and G2/M phase cell cycle arrest [5] and inhibits human leiomyoma cell proliferation via crosstalk between integrin αvβ3 and insulin-like growth factor 1 receptor (IGF-1R) [6]. Resveratrol also suppresses the signal transducer and activator of transcription 3 (STAT3) signaling pathway and inhibits proliferation of high-glucose-exposed HepG2 cells, partly via sirtuin-1 (SIRT1) [7]. However, the molecular mechanisms underlying the antitumor effects of resveratrol in HCC still need further exploration.

MARCH1, an E3 ubiquitin ligase with a membrane-associated RING-CH domain, can regulate congenital immunity Preliminary [8]. Research in our lab found that MARCH1 is significantly higher expressed in HCC tissues and cells. MARCH1 knockdown by small interfering RNA (siRNA) could induces the apoptosis, inhibit the proliferation, migration, and invasion of HCC cells [9]. We have shown that MARCH1 is a tumor promoter that regulates the phosphoinositide-3-kinase/protein kinase B (PI3K/AKT)/β-catenin signaling pathway and might be a potential molecular therapeutic target in HCC [10].

This study determined that resveratrol not only inhibits the biological functions of HCC cells (consistent with previous reports) but also downregulates MARCH1 expression dose-dependently. We also analyzed whether resveratrol elevates the expression of phosphatase and tensin homolog deleted on chromosome 10 (PTEN) and inhibits phospho–protein kinase B (P-AKT) levels. Overall our results demonstrated that resveratrol may through reduced MARCH1 regulated of PTEN/AKT pathway, inhibited expression of Bcl-2 and VEGF, in turn exerted anti-HCC effects

## RESULTS

### Resveratrol suppressed HCC cell viability via down-regulated MARCH1 of HepG2 and Hep3B cells

**Figure 1:**
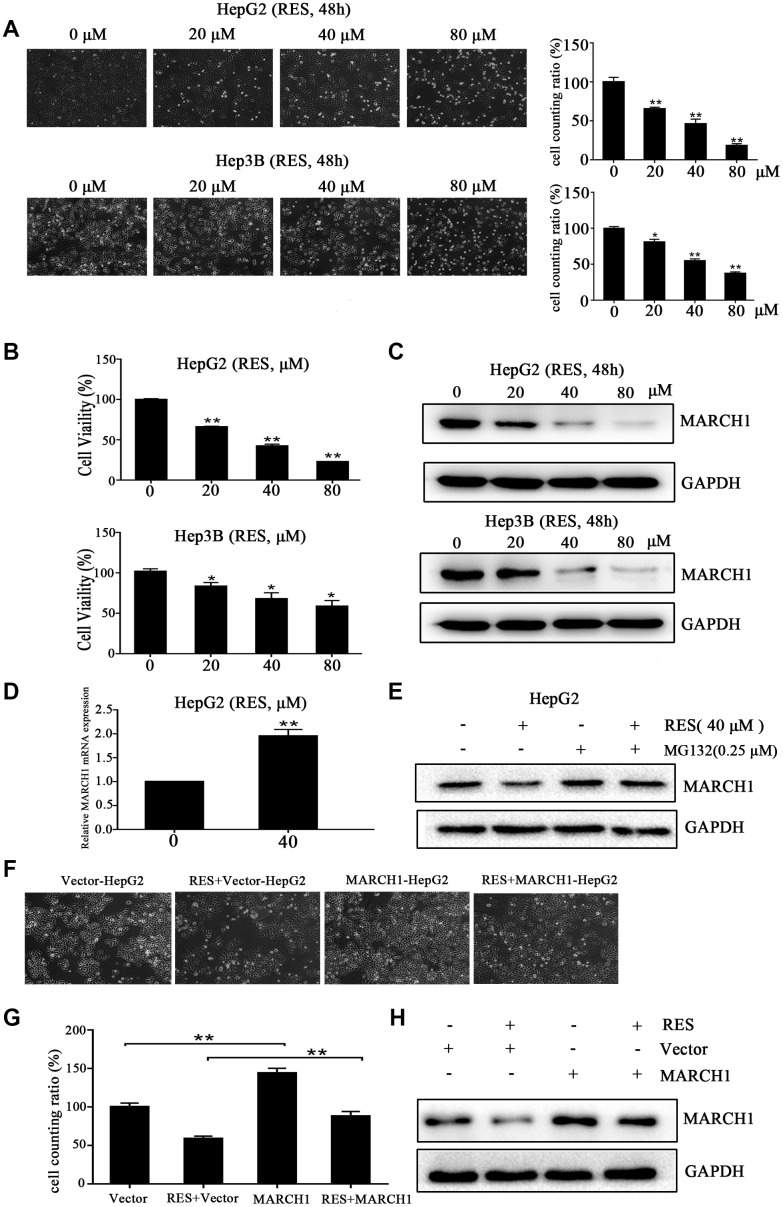
**Resveratrol inhibits HCC cell viability.** (**A**) Representative images of HepG2 and Hep3B cells treated with different concentrations of resveratrol for 48 h. (**B**) The cytotoxicity of HepG2 and Hep3B cells treated with resveratrol was estimated by CCK-8 assays. HepG2 and Hep3B cells were treated with different concentrations of resveratrol for 48 h. The absorbance at 450 nm is detected. (**C**) Western blot analysis showed that MARCH1 expression decreased after resveratrol treated for 48 h in both HepG2 and Hep3B cells. GAPDH was also detected as the loading control. (**D**) HepG2 cells were treated with the indicated dose of resveratrol for 24h and then analyzed the transcription level of MARCH1. MARCH1 mRNA levels were normalized to GAPDH. (**E**) HepG2 cells were pretreated with 0.25μM MG132 for 24h, then analyzed the expression of MARCH1. (**F**, **G**) HepG2 cells were treated with the indicated dose of empty vectors and overexpression plasmids for 48h, then incubation with resveratrol for 24h. (**H**) The expression of MARCH1 were detected. All values represent the mean ± SD. **P < 0.01; *P < 0.05.

We used CCK-8 assays to validate the potential cytotoxic effects of resveratrol on HCC cell lines (HepG2 and Hep3B). Treatment of HepG2 and Hep3B cells with 20, 40, and 80 μM resveratrol for 48 h affected the number and state of cells (Figure 1A). MARCH1 expression was significantly down-regulated (Figure 1C). In addition, resveratrol was evidently cytotoxic to HepG2 and Hep3B cells dose-dependently by (Figure 1B). After resveratrol treatment for 48 h, the mean inhibitory concentration (IC_50_) was 32.33 and 113.5 μΜ for HepG2 and Hep3B cells, respectively. Interestingly, the expression of MARCH1 in mRNA was increased slightly (Figure 1D) after treated with the indicated dose of resveratrol for 24h in HepG2 cells. Then, treated cells with resveratrol and with or without MG132, a proteasome inhibitor, and analyzed the results by Western blot. Resveratrol treatment led to a decreased expression of MARCH1, treatment with MG132 blocked the decreased of MARCH1 induced by resveratrol. (Figure 1E). These results suggested that resveratrol promoted proteasome –mediated degradation of MARCH1. To further validate that anti-tumor effect of resveratrol is mediated by inhibition of MARCH1, transfected HepG2 cells with empty vector and MARCH1 overexpression plasmids, and then treated with or without resveratrol for 24h. The data shows that combined with MARCH1 overexpression plasmids group would partially cancel anti-tumor effect by resveratrol (Figure 1F, 1G). Then, the protein level were analyzed by Western blot, the results were consistent with the cell phenotype (Figure 1H). These data indicated that inhibition of cell viability induced by resveratrol might be involved in down-regulation of MARCH1 expression.

### Resveratrol induced apoptosis and inhibited colony formation of HCC cells

**Figure 2:**
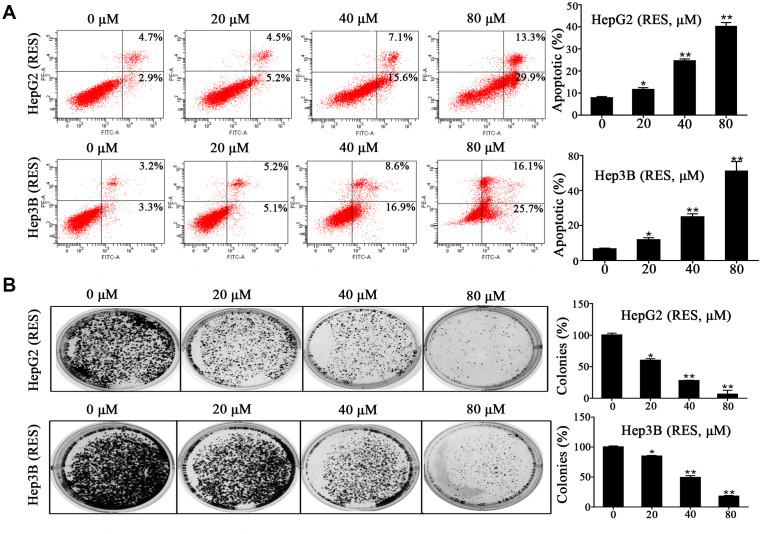
**Resveratrol induces HCC cell apoptosis.** (**A**) HepG2 and Hep3B cells were treated with different concentrations of resveratrol for 48 h, followed by analysis of cell apoptosis by flow cytometry. Representative scatter plots are shown. The percentage of apoptosis was calculated. (**B**) HepG2 and Hep3B cells were seeded in 6-well plates and treated with resveratrol for 24 h, and the medium was changed with fresh medium for 12 days to form colonies. Colonies were fixed and stained. All values represent the mean ± SD. **P < 0.01; *P < 0.05.

Experiments on apoptosis were performed to determine the molecular mechanism underlying resveratrol’s effect on cell growth. HepG2 and Hep3B cells were treated with various concentrations of resveratrol (0, 20, 40, and 80 μM) for 48 h. Flow cytometric analysis showed that resveratrol treatment significantly increased the rate of apoptosis compared to control cells. The number of apoptotic Hep3B cells increased by 1.8-, 3.8-, and 7.8-fold, while the number of apoptotic HepG2 cells increased by 1.5-, 3.2-, and 5.2-fold in response to 20, 40, and 80 μM resveratrol, respectively (Figure 2A). We also assessed the effect of resveratrol on the colony formation ability of HepG2 and Hep3B cells. The numbers of colonies significantly decreased in resveratrol-treated cells compared to control cells (Figure 2B). These data demonstrated that cell growth inhibition contributes to resveratrol-induced HCC cell apoptosis.

### Resveratrol inhibited the migration and invasion of HCC cells

**Figure 3:**
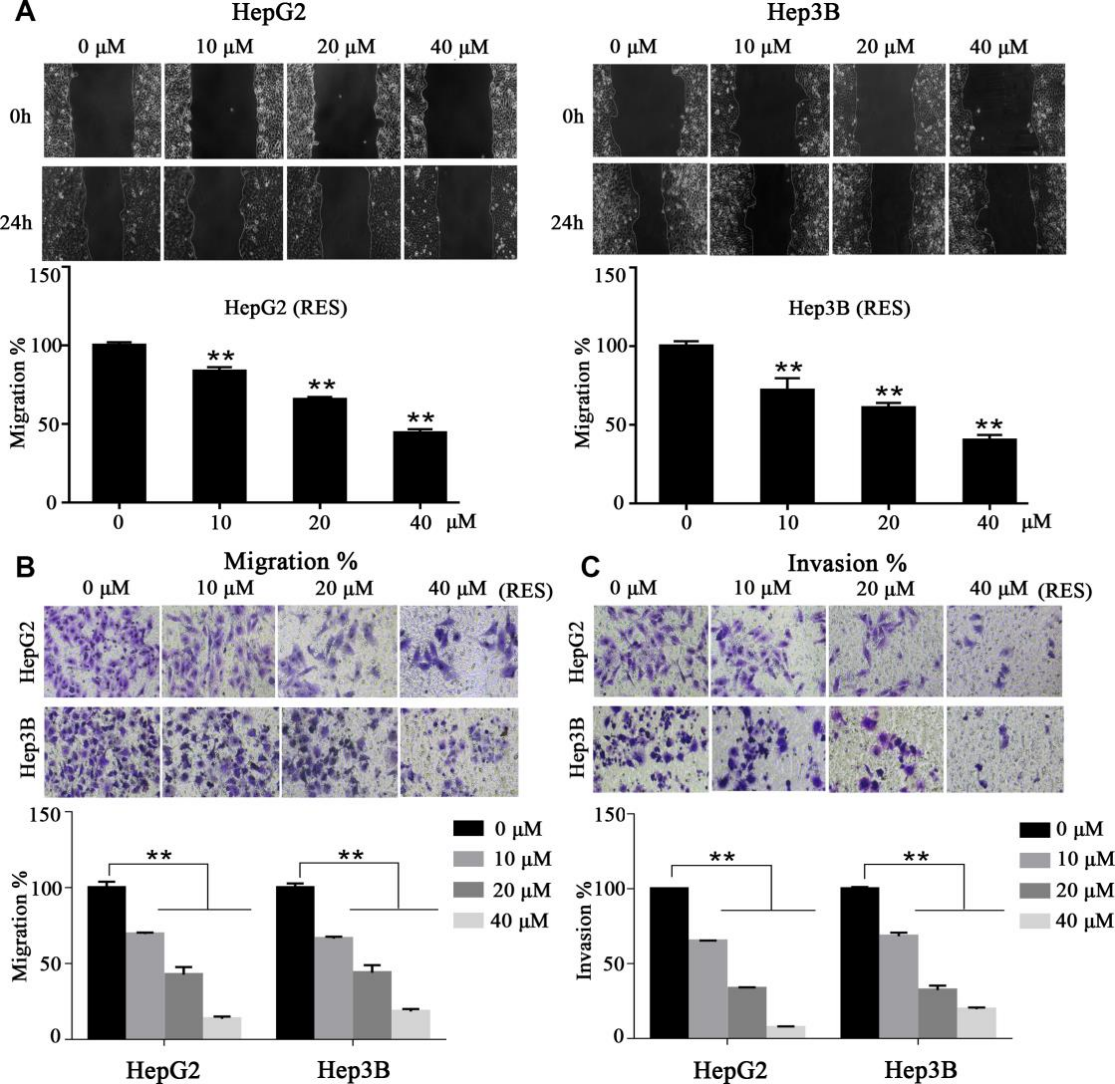
**Resveratrol inhibits HCC cell motility.** (**A**) In a wound-healing assay, HepG2 and Hep3B cells were treated with different concentrations of resveratrol for 24 h. The wound area was measured at indicated times. Representative images are shown. (**B**, **C**) Cell tran-swell assays (with or without a Matrigel coating). HepG2 and Hep3B cells were seeded in the upper chamber and cultured in serum-free medium containing resveratrol. After 24 h, the membrane was stained with crystal violet solution and photographed. The results of quantification analysis are presented. All values represent the mean ± SD. **P < 0.01; *P < 0.05.

To investigate whether resveratrol affects cell motility, we performed wound-healing and trans-well assays within 24 h and with low concentrations of resveratrol in order to avoid cell proliferation and apoptosis. Wound-healing assays showed that resveratrol treatment impairs HCC cells’ motor ability compared to control cells. The migration rate decreased by ~about 28%, 39%, and 60% in HepG2 cells and by ~16%, 35%, and 56% in Hep3B cells with 10, 20, and 40 μM of resveratrol, respectively (Figure 3A). In addition, trans-well assays showed that resveratrol treatment sharply decreased the invasiveness of HepG2 and Hep3B cells. The rate of migration of HepG2 and Hep3B cells decreased to 14% and 18%, respectively, compared to control cells with 40 μM (maximum concentration) (Figure 3B). In addition, the invasion percentage of HepG2 and Hep3B cells decreased to 7% and 19%, respectively, compared to control cells with 40 μM (maximum concentration) (Figure 3C). These results suggested that resveratrol inhibits HCC cell migration and invasion in vitro.

### Resveratrol decreased expression of MARCH1 and Its related genes

**Figure 4:**
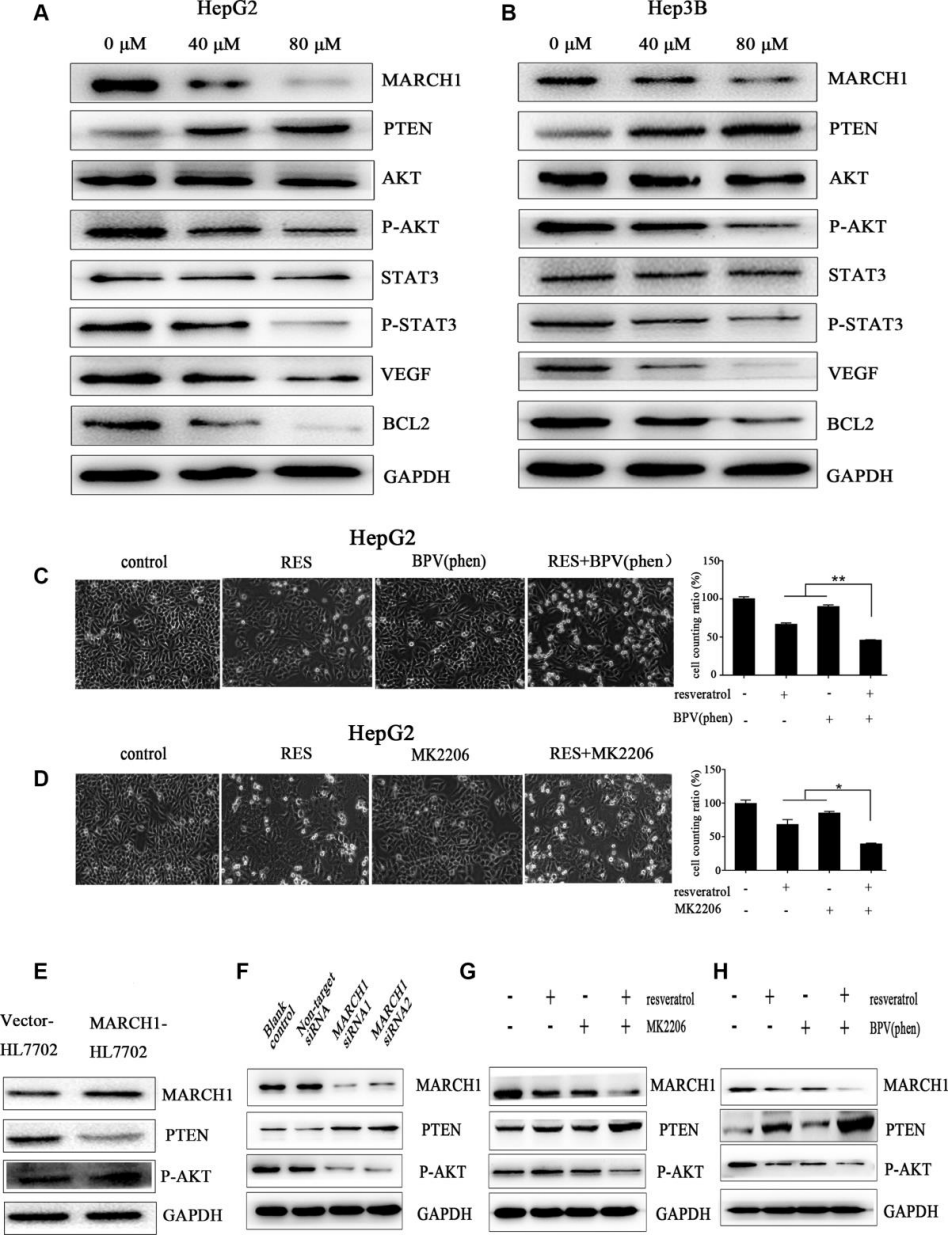
**Resveratrol could down-regulate MARCH1 expression via PTEN–STAT3 signaling.** (**A**, **B**) HepG2 and Hep3B cells were treated with different concentrations of resveratrol for 48 h, and the level of protein expression was analyzed by western blotting. MARCH1 and p-AKT levels significantly drastically decreased, PTEN levels increased, and downstream protein molecules also significantly decreased. (**C**, **D**) HepG2 cells were treated with inhibitors MK2206 and BPV(phen). The combination of resveratrol and inhibitors significantly inhibited cell survival compared to resveratrol alone. (**E**) Overexpression of the MARCH1 protein with empty vectors and overexpression plasmids in the human HL7702 cells. (**F**) HepG2 cells were infected with indicated concentrations of siRNA for 72 h. MARCH1 expression significantly decreased, while PTEN expression increased. (**G**, **H**) HepG2 cells were pretreated with an inhibitor for 12 h and then combined with resveratrol for 48 h. MARCH1 expression decreased even more compared to resveratrol alone. GAPDH was also detected as a loading control.

Previous studies have verified that PTEN/AKT has been inhibited the process of tumor initiation, promotion, and progression. To explain the molecular mechanism underlying inhibition of the biological functions of HCC cells by resveratrol, we employed western blotting assays. As show in Figure 4A, 4B, resveratrol treatment significantly decreased MARCH1 and p-AKT expression, increased PTEN expression, and significantly decreased the expression of downstream protein molecules, such as VEGF, transcription factor p-STAT3, and the anti-apoptotic gene *Bcl2* in both HepG2 and Hep3B cells. To explore the relationship between MARCH1, PTEN, and p-AKT, we used overexpressed plasmids, siRNA and small-molecule inhibitors with or without resveratrol treatment of HepG2 cells. Results showed that the combination of resveratrol and inhibitors significantly inhibited cell survival compared to resveratrol alone, which was also confirmed by western blotting assay (Figure 4C, 4D). Furthermore, the expression of PTEN were decreased and the level of P-AKT increased after forced expression of MARCH1 (Figure 4E). Also, MARCH1 knockdown by siRNA increased PTEN levels, which was in accordance with resveratrol treatment (Figure 4F). HepG2 cells were pretreated for 12 h with MK2206 and BPV(phen) as inhibitors of p-AKT and PTEN, respectively, and then combined with resveratrol. Results showed that the protein level of MARCH1 decreased even more compared to resveratrol alone (Figure 4G, 4H). In summary, these results indicated that resveratrol might ameliorate the progression of HCC through PTEN-AKT signaling via down-regulation of MARCH1 expression in vitro.

### The expression of PTEN mRNA were increased

HepG2 cells were treated with the indicated dose of resveratrol for 24h and then analyzed the transcription level of PTEN. The mRNA level of PTEN was up-regulated after treatment with resveratrol. To demonstrate how MARCH1 regulates PTEN, HepG2 cells were infected with indicated concentrations of siRNA for 48 h. Then the mRNA MARCH1 expression significantly decreased, while mRNA PTEN expression increased. To sum up, after the treatment of resveratrol or knockdown of MARCH1 by siRNA of HepG2 cells respectively stimulated the up-regulation of PTEN at the transcriptional level consistent with the protein level (Supplementary Figure 1A, 1B).

### Resveratrol significantly inhibits tumor growth in vivo

**Figure 5:**
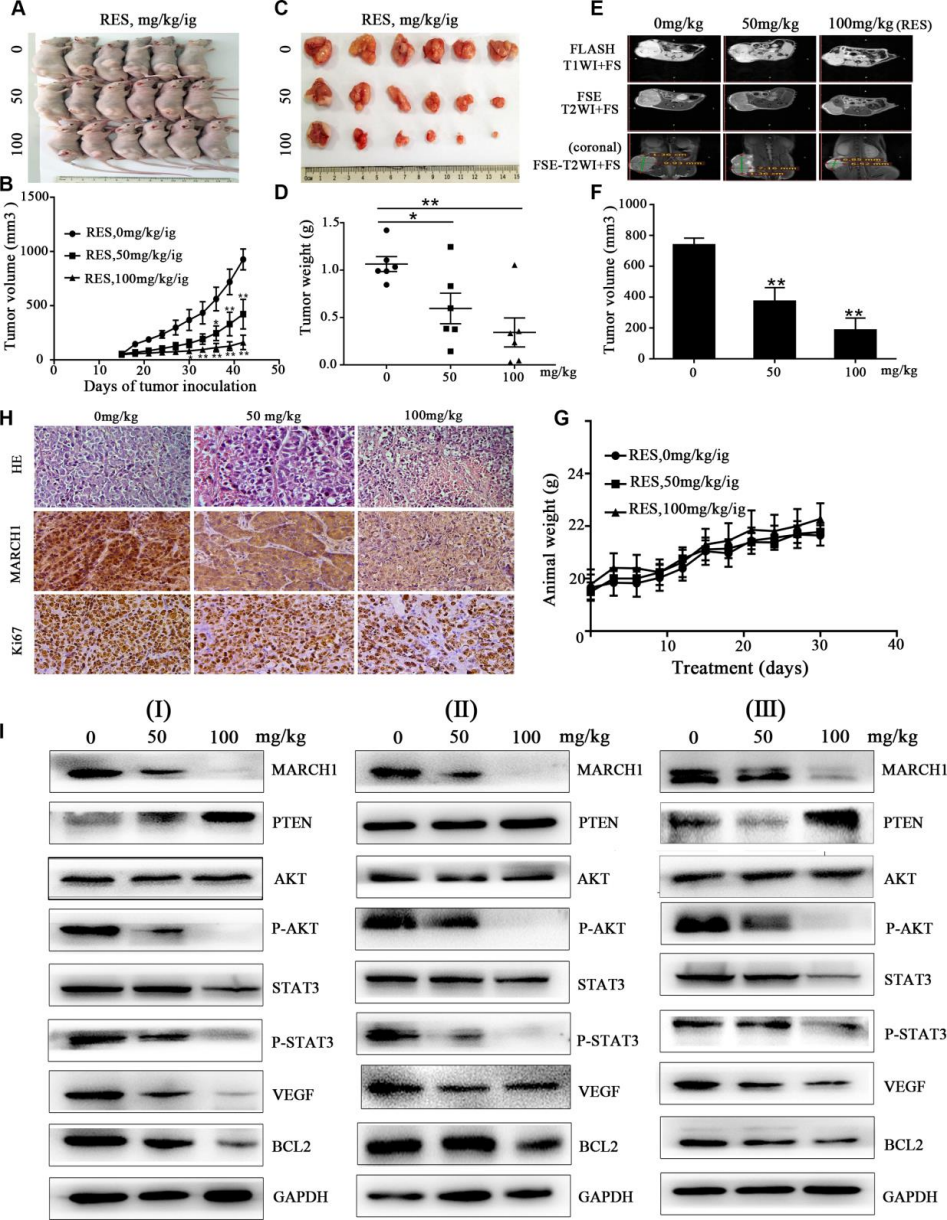
**Resveratrol inhibits tumor growth in HepG2 mouse models.** (**A**–**C**) HepG2 cells were inoculated into the back of BALB/c nude mice, near the right hind leg. The mice were treated with resveratrol (0, 50, and 100 mg/kg/ig). Representative images of the tumor are shown, and the tumor volume growth curves of resveratrol are plotted. (**D**) Tumor weight after resveratrol treatment. (**E**) Representative images of mice MRI. (**F**) Average volume of tumor measured on coronal T2-weighted MRI after resveratrol treatment. (**G**) The changes in animal weight are shown during resveratrol treatment. (**H**) Tumor tissue slides were stained with H&E and IHC performed. (**I**) Levels of MARCH1, PTEN, p-AKT, p-STAT3, VEGF, and Bcl2 in tumors treated with resveratrol were analyzed by western blotting. GAPDH was also detected as a loading control. All values represent the mean ± SD. **P < 0.01; *P < 0.05.

To further confirm the antitumor effects of resveratrol in HCC, we used established xenograft models; we inoculated HepG2 cells into the back of BALB/c nude mice, near the right hind leg. The mice treated with resveratrol at the indicated concentration showed significant inhibition of tumor volume and tumor weight dose-dependently (Figure 5A–5D). MRI was used to analyze the therapeutic effects of resveratrol. As shown in Figure 5E, 5F, on coronal T2-weighted MRI, the tumor volume after resveratrol treatment was significantly decreased, which was consistent with the measurement using a digital vernier caliper. However, the weight of the three groups of mice was not statistically significant during the treatment period (Figure 5G). In addition, hematoxylin and eosin (H&E) staining of tissues treated with resveratrol showed looser cell spacing and much more apoptotic corpuscles and tumor necrosis compared to control groups. IHC showed that resveratrol treatment decreases MARCH1 expression. Ki67 is a nuclear protein strictly associated with cell proliferation, which is present in all active phases of cells but is absent in resting cells, making it a proliferation marker [11]. We observed that resveratrol treatment decreased the number of stained nuclei dose-dependently (Figure 5H). In addition, the protein from tumor tissue was used to verify the signal pathway by western blotting assay. Consistent with in vitro studies, the expression levels of key proteins, such as MARCH1 and p-AKT, significantly decreased, while PTEN levels increased (Figure 5I). Taken together, these results showed that resveratrol significantly inhibits tumor growth in vivo.

**Figure 6:**
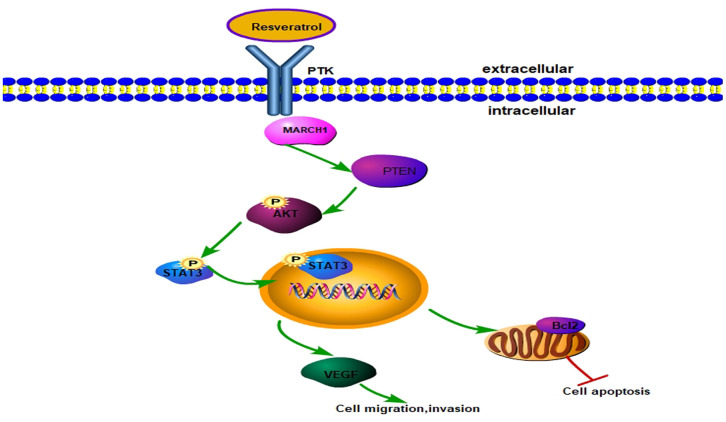
**Model for resveratrol regulating MARCH1 expression.** Resveratrol decreased MARCH1 expression, increased PTEN expression, and decreased AKT phosphorylation, STAT3 phosphorylation. On the whole, resveratrol inhibits the malignant progression of HCC via MARCH1-induced regulation of the PTEN/AKT/STAT3 signaling pathway. The expression of downstream effectors VEGF and Bcl2 decreased.

The Potential Signal Pathway against HCC with Resveratrol though Regulating MARCH1.Resveratrol could reduce the level of MARCH1 then regulates the PTEN/AKT signaling pathway thereby affecting cell proliferation, apoptosis, migration, and invasion, and the signal pathway diagram is plotted (Figure 6).

## DISCUSSION

Resveratrol is a common, naturally occurring polyphenol derived from many dietary substances and has numerous biological activities [4]. Studies have revealed that resveratrol inhibits the cellular effects of carcinogenesis, which is the stage of tumor initiation, promotion, and progression [12]. Resveratrol might be a promising therapeutic and chemotherapeutic preventive agent against various types of cancer, including breast, colorectal, skin, and liver cancers [13]. Resveratrol could interact with a wide range of proteins, including receptors, kinases, and signaling molecules, thus controlling cell growth, apoptosis, metastasis, and angiogenesis [14, 15]. This study illustrated that resveratrol down-regulates MARCH1 expression, increases PTEN expression, decreases AKT phosphorylation and STAT3 nuclear translocation, inhibits of VEGF and Bcl2 expression, and subsequently affects the biological hallmarks of HCC cells. These results demonstrated a novel molecular mechanism underlying resveratrol treatment of HCC.

MARCH1 is an E3 ubiquitin ligase. Ubiquitination is involved in the regulation of protein degradation and almost all life activities, including the cell cycle, gene expression, and signal transduction [16]. A preliminary study in our lab found that MARCH1 expression increases in HCC cell lines (HepG2 and Hep3B). We also confirmed that MARCH1 can promote the development of HCC, so it might be a potential molecular therapeutic target. In this report, found that resveratrol treatment significantly down-regulates MARCH1 expression in HCC cells dose-dependently and obviously induces inhibition of cell proliferation and increases apoptosis. Due to the heterogeneity of cancer, it is difficult to find a single target for cancer therapy. In addition, mono-targeted drugs usually accompany with several side effects if taken for a long time. Some studies have reported that resveratrol regulates multiple signaling pathways, so resveratrol antitumor treatment might affect multi-target molecules [17, 18]. Based on our previous reports and present findings, we supposed that MARCH1 might be a novel therapeutic target of resveratrol. Also the down-regulation of MARCH1 expression is an important molecular mechanism of resveratrol’s anticancer efficacy in HCC.

Genetic mutation or deletion of *PTEN*, a tumor suppressor gene, is commonly noted in human cancers and might be involved in the development and progression of HCC [19]. PTEN is a lipid phosphatase, which can dephosphorylate proteins, negatively regulating the PI3K/AKT signaling pathway, which is one of the mostly universal dysregulated signaling pathways in cancers [20]. Phosphorylated AKT (p-AKT) represents the active form, and aberrant overexpression of p-AKT is a common feature in cancers [21]. Resveratrol reverses doxorubicin resistance via modulation of the PTEN/AKT signaling pathway in gastric cancer [22]. Moreover, resveratrol treatment can increase PTEN expression, leading to decreased p-AKT expression and proliferation index in prostate cancer [23]. Whether the PTEN/AKT signaling pathway is involved in anti-HCC treatment with resveratrol has not been reported. In this study, resveratrol treatment up-regulated PTEN expression in HCC cells dose-dependently. In addition, expression of AKT phosphorylation decreased with increasing resveratrol concentration. Thus, the PTEN/AKT signaling pathway exerts a significant effect on anti-HCC treatment with resveratrol. AKT is a crucial signaling hub with a number of downstream target substrates [24]. In addition, AKT regulates several transcription factors, including fork-head box transcription factor class O (FOXO), cyclic adenosine monophosphate (cAMP)-response element-binding protein (CREB), and STAT3 [25–27]. STAT3 is an important transcription factor. Comparison of organellar localization after gene activation shows that AKT remains in the cytoplasm, while STAT3 is mainly located in the nucleus [32]. Previous reports have shown that AKT acts upstream of STAT3 in some cancers, regulating cell viability, invasion, and migration [28, 29]. In this study, we observed that resveratrol treatment markedly inhibits STAT3 phosphorylation in HCC.VEGF expression is usually high in HCC, and VEGF is one of the major regulators in angiogenesis [30]. As a necessary process for invasion and metastasis, tumor angiogenesis plays an important role in cancer progression, meanwhile, tumor metastasis is believed to be the leading cause of cancer-related death [31]. STAT3 is closely associated with VEGF, that is, STAT3 directly binds to the VEGF promoter and mediates VEGF transcription [32]. In this study, VEGF expression was down-regulated with increasing concentration of resveratrol. Bcl2 is an anti-apoptotic protein of the Bcl-2 family that modulates survival in various cancers [33]. We found that resveratrol treatment decreased Bcl2 expression dose-dependently. This and previous studies have suggested that resveratrol interacts with a wide range of proteins; however, it is worth investigating whether there is cascading effect and reciprocal regulation among these proteins.

To explore the exact molecular mechanism underlying resveratrol treatment of HCC, we applied the siRNA-induced MARCH1 knockdown. PTEN expression increased and the expression of p-AKT were down-regulated in the MARCH1 siRNA group compared to the non-target group. Meanwhile, compared with the empty vector-HL7702, the expression of PTEN was decreased and p-AKT was up-regulated in the overexpression MARCH1-HL7702 cells. These results indicated that resveratrol inhibits the malignant progression of HCC via MARCH1 regulation of the PTEN/AKT signaling pathway. To further prove that resveratrol inhibits the malignant progression of HCC via MARCH1-induced regulation of the PTEN/AKT signaling pathway, the PTEN inhibitor BPV(phen) and the p-AKT inhibitor MK2206 combined with resveratrol were used to treat HepG2 cells and then analyzed the changes and correlation of these related genes expression. These present results exhibited the synergy with resveratrol, the MK2206 and resveratrol combination group had lower number of cells than single treatment group. Under the action of MK2206 inhibitor, the combination group had lowest expression of MARCH1, however, the level of PTEN was highest. Similar results were showed with BPV(phen), the level of p-AKT and MARCH1 was lowest in combination group. According to the above results, we suspect that resveratrol demonstrated anti-cancer effect may through the interaction of MARCH1, PTEN, and p-AKT, or say cyclic reciprocal impact, then transmit signals to downstream molecules, change the biological phenotype of cells. However, the more detailed and precise proteins interactions with each other remains to be studied. At the same time, resveratrol is safe up to a concentration of 5 g/d [34]. In this study, the concentration of 100mg/kg/2days of resveratrol could effectively inhibit cancer growth yet with minimal toxicity to normal cells.

In conclusion, resveratrol down-regulate MARCH1 expression, and MARCH1 regulates the PTEN/AKT signaling pathway, affecting the biological functions of HCC cells, that is, it inhibits HCC cell growth and induces apoptosis. This is a new potential mechanism for use of resveratrol as treatment of HCC and might contribute to the early clinical application of resveratrol in HCC.

## MATERIALS AND METHODS

### Reagents and antibodies

Resveratrol was purchased from Solarbio Life Sciences (R8350; Beijing, China). It was dissolved in dimethyl sulfoxide (DMSO) to an appropriate concentration and then stored at –20°C until use. The AKT inhibitor MK2206 (HY-10358) was purchased from MedChemExpress (MCE, NJ, USA). The PTEN inhibitor potassium bisperoxo (1,10-phenanthroline) oxovanadate (BPV(phen)) (SML-0889) was purchased from Sigma-Aldrich (St. Louis, MO, USA). Anti-MARCH1 (bs-9335) antibody was obtained from Bioss (Beijing, China). In addition, anti-PTEN (22034-1-AP), AKT (60203-2-IG), P-AKT (S473; 66444-1-AP), B-cell lymphoma 2 (Bcl2; 12789-1-AP), vascular endothelial growth factor (VEGF; 1900 3-1-AP), STAT3 (10253-AP), and glyceraldehyde 3-phosphate dehydrogenase (GAPDH; 10494-1-AP) antibodies were purchased from Proteintech (Wuhan, China). Anti-p-stat3 (S727; ab3214) antibody was purchased from Abcam (Cambridge, UK), while horseradish peroxidase (HRP)-conjugated secondary antibodies for western blotting (ZB-2301 and ZB-2305) and immunohistochemistry (IHC) (PV6000), DAB (ZLI-9017), goat serum (ZLI-9022), and Ki67 (ZM-0167) were obtained from ZSGB-BIO (Beijing, China).

### Cell culture

HCC cell lines HepG2 and Hep3B were purchased from Cell Line Bank, Chinese Academy of Science (Shanghai, China). The cells were maintained in Dulbecco’s modified Eagle medium (DMEM) containing high glucose (HyClone, Logan, UT, USA), 10% fetal bovine serum (FBS; Gibco, Waltham, MA, USA), 100 μg/mL of streptomycin, and 100 U/mL of penicillin and incubated in a humidified atmosphere of 5% CO_2_ at 37°C.

### Quantitative real-time RT-PCR

Real-time (RT)PCR primer designed and synthesized by Takara. The primer sequences for human MARCH1 were F:5’-CTGCTGTGAGCTCTGCAAGTATGA-3’; R: 5’-TACGTGGAATGTGACAGAGCAGAA. The primer sequences for human PTEN were F: 5’-TGAAGTGAGGCTTGTAGTCATGG-3’, R:5’-CATT TGGACAACTGGATAGAGTAGG-3’. The primer sequences for human GAPDH were F: 5’-GCACCGTCAAGGCTGAGAAC-3’; R: 5’-TGGTGAAGACGCCAGTGGA-3’. Briefly, total RNA was extracted from HepG2 cells using TRIZOL, and the cDNAs were reverse transcribed using the Takara reverse-transcription kit (RR047A.Takara).The genes were amplified and detected by real-time PCR using Tli RNaseH Plus(RR820A,Takara). Refer to the factory manual for specific steps. Relative quantification with the comparative threshold cycle (Ct) was done using the Ct method.

### Gene silencing and transfection

Two different siRNA sequences targeting different sites ofMARCH1 mRNA were designed and provided by Genepharma (Shanghai, China). The sequences for the MARCH1 siRNA-1, the sense sequence 5′- CAGGAGGUCUUGUCUUCAUTT-3′; the antisense sequence 5′-AUGAAGACAAGACCUCCUGTT-3′. For siRNA-2, the sense sequence 5′-GGUAGUGCCUGUACCACAATT-3′; the antisense sequence 5′-UUGUGGUACAGGCACUACCTT-3′.

The non-target siRNA was the sense sequence 5′-UUCUCCGAACGUGUCACGUTT-3′; the antisense sequence 5′-ACGUGACACGUUCGGAGAATT-3′. The over-expressed plasmid was originally stored in the laboratory.

### Cell proliferation assay

The antiproliferative effects of resveratrol on HCC were determined using Cell Counting Kit-8 (CCK-8; Biosharp, Beijing, China). Briefly, HepG2 and Hep3B cells were seeded in 96-well plates at a density of 5 × 10^3^ cells/well overnight, treated with indicated concentrations of resveratrol for 48 h, and incubated with CCK-8 reagent for 1 h at 37°C, avoiding light. Absorbance of the samples was recorded at a wavelength of 450 nm busing a SpectraMax M2 microplate reader (Molecular Devices, Shanghai, China).

### Colony formation assay

HepG2 and Hep3B cells were seeded in 6-well plate at a density of 8000 cells/well. On the following day, the cells were treated with resveratrol for 24 h, and the medium was changed with fresh medium for 12 days to form colonies. The colonies were fixed and stained with 0.1% crystal violet solution (Solarbio, Beijing, China), and colonies with >50 cells were counted.

### Cell apoptosis assay

HepG2 and Hep3B cells were managed using the fluorescein isothiocyanate (FITC)–annexin V and propidium iodide (PI) apoptosis detection kit (KeyGEN Biotech, Nanjing, China) according to the manufacturer’s instructions. Briefly, HepG2 and Hep3B cells were incubated with resveratrol in a 6-well plate for 48 h. The cell apoptosis rate was determined using a FACSCanto flow cytometer (Becton Dickinson, Franklin Lakes, NJ, USA).

### Wound-healing assay

HepG2 and Hep3B cells were seeded in a 6-well plate and incubated with DMEM until they reached 90% confluence. Scratches were made using a 10 μL pipette tip, the cells were washed with phosphate-buffered saline (PBS), and the medium was changed with fresh medium and 1% FBS to inhibit cell proliferation. The scratch areas were photographed at indicated times using an Olympus TL4 photomicroscope (Olympus, Japan). The wound areas were measured using Image-Pro Plus 6.0, and wound-healing mobility was calculated according to the change in wound size.

### Transwell assay

HCC cell migration and invasion were measured in a 24-well transwell membrane chamber with 6.5 mm inserts and 8.0-μm-pore polycarbonate membranes (3422; Corning Inc., Corning, NY, USA). Briefly, for the invasion assay, the transwell membrane chamber was covered with Matrigel (356234; Corning) and incubated for 4 h at 37°C. Then, the excess Matrigel was removed gently. HepG2 and Hep3B cells were seeded in the upper chamber at a density of 5 × 10^4^ cells/well and cultured in serum-free medium containing resveratrol. The lower chamber contained complete medium with 20% FBS. After 24 h, the cells remaining on the upper side of the membrane that was untraversed were be removed using a wet cotton swab. The membrane was fixed with 4% paraformaldehyde (PFA) for 20 min and stained with 0.1% crystal violet solution for 10 min. Excess dye was washed off, and the cells were photographed under a microscope at 400Х magnification (Canon, China).

For the cell migration assay, there was no need for the Matrigel coating. The other steps were the same as before.

### Xenograft model and tumortherapy

All animal protocols were approved by the Institutional Animal Care and Use Committee of Binzhou Medical University, China (SCXK2018-0008). Female BALB/c nude mice aged 4 weeks and weighing 18–22 g were purchased from GemPharmatech Co. Ltd (Nanjing, China) and bred in a specific pathogen-free (SPF)-grade laboratory. The mice were allowed ~1 week to adapt to their new environment, which was a 12/12 h light/dark cycle in a temperature-controlled room. Briefly, 1 × 10^7^ HepG2 cells were suspended in 150 μL of PBS and subcutaneously inoculated into the back of the mice, near the right hind leg. After 2 weeks, tumor volumes reached ~50–70 mm^3^. The mice were randomly divided into three groups. Resveratrol was dissolved in filtered corn oil and intragastrically administered at concentrations of 0, 50, and 100 mg/kg every 2 days. The tumor volumes were measured using a digital caliper once every 2 days and calculated as (*A* × *B*^2^)/2, where *A* and *B* are the longest and shortest diameter of the tumor, respectively. When the tumor size of the control group reached ~10–15 mm, all mice were scanned for magnetic resonance before they were euthanized. The tumors were harvested and divided into two parts, one for western blotting and the other for IHC.

### Western blotting

HepG2 and Hep3B cells were lysed using ice-cold radioimmunoprecipitation assay (RIPA) lysis buffer (P0013B; Beyotime, Shanghai, China) containing protease inhibitors phenylmethanesulfonyl fluoride (PMSF; Beyotime) and phosSTOP (Roche, Indianapolis, IN, USA). The cells were split on ice for 30 min and then centrifuged for 20 min at 12,000 × *g* and 4°C to remove debris. The concentration of protein extracts was determined using a bicinchoninic acid (BCA) protein assay kit (Beyotime) and boiled in 5Х loading buffer for 8 min. Total protein (25 ug) was loaded per lane and resolved by sodium dodecyl sulfate–polyacrylamide gel electrophoresis (SDS-PAGE) and then transferred to polyvinylidene difluoride (PVDF membrane) (ISEQ00010; Merck Millipore, Germany). The PVDF membrane was incubated overnight with a primary antibody at 4°C and secondary antibodies for 2 h at room temperature and electroluminescence was detected using a super-enhanced chemiluminescence (ECL) kit (Novland, Shanghai, China). Band quantification was performed using ImageJ. GAPDH was used as the internal control.

### Immunohistochemical staining

All samples were fixed in 4% PFA and embedded in paraffin. Tissue slices (5-μm-thick) were dewaxed in xylene, dehydrated in ethanol and block endogenous peroxidase for 10 min, and incubated in goat serum for 30 min. The slides were incubated with primary antibodies targeting ki67, MARCH1 (1:200 YT2642; Immunoway) overnight at 4°C and then with the HRP-conjugated secondary antibody for 30 min at 37°C. Finally, the sections were visualized under a Leica light microscope (Leica Microsystems, Wetzlar, Germany) after staining with DAB, a chromogenic reagent.

### Magnetic resonance imaging

All magnetic resonance imaging (MRI) examinations were performed using a high-field 7.0 Tesla Bruker BioSpec 70/20 USR MRI system (Bruker, Karlsruhe, Germany). MRI of the mice was performed in the prone position, using a nonmagnetic stereotactic and cylindrical surface wrist coil for signal detection. The mice were anesthetized with 1%–2% inhaled isoflurance (Ruiward Life Technology Co. Ltd., Shenzhen, China) in the following sequence: T2 fast spin echo (FSE) sequence repetition time (TR) = 1986.57 ms; echo time (TE) = 34.37 ms; slice thickness (ST) = 1 mm. The total acquisition time was ~20 min.

### Statistical analysis

Statistical analysis was performed using SPSS Statistics 17.0 (IBM Corporation, Chicago, IL, USA) and PRISM 7.0 (GraphPad Prism, San Diego, CA, USA). Two-tailed Student’s *t*-test, one-way analysis of variance (ANOVA; for >2 groups), and two-way ANOVA were used to analyze the defenses of volume between mice. *P* < 0.05 was considered statistically significant.

## Supplementary Material

Supplementary Figure 1
