# Supplementary material for: Resveratrol inhibits the malignant progression of hepatocellular carcinoma via MARCH1-induced regulation of PTEN/AKT signaling
Source: Aging (Albany NY). 2020 Jun 12;12(12):11717–31. doi: 10.18632/aging.103338 (PMC7343503; doi:10.18632/aging.103338)
Supplement: Supplementary Figure 1 [file aging-12-103338-s001..pdf]

SUPPLEMENTARY FIGURE

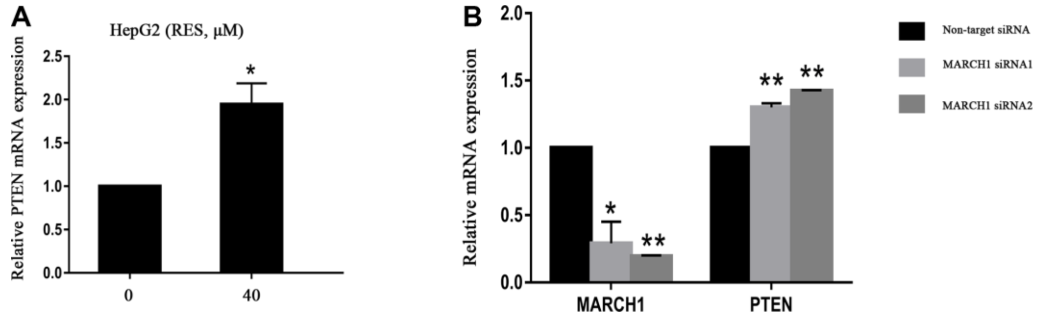

Supplementary Figure 1. The expression of PTEN mRNA were demonstrated. (**A**) HepG2 cells were treated with the indicated dose of resveratrol for 24h and then analysed the transcription level of PTEN. (**B**) HepG2 cells were infected with indicated concentrations of siRNA for 48 h. Then the mRNA MARCH1 expression significantly decreased, while mRNA PTEN expression increased.
